# 

**Published:** 1887-09-17

**Authors:** 


No. 51, Vol. II.

September 17th, 1887.

Registered at the General Post Office as a Newspaper.]

Per Annum, 6s. 6d., post free.
Monthly Parts, 6d.; post free, 7½d.
Ditto per Ann., 7s. 6d. post free.

